# The HSP-90 co-chaperone CDC-37 regulates oomycete immunity in *C. elegans* through a membrane-associated pseudokinase

**DOI:** 10.64898/2026.07.31.741986

**Published:** 2026-07-31

**Authors:** Franziska Trusch, Michalis Barkoulas

## Abstract

Innate immune responses depend on rapid and tightly regulated signalling to ensure effective defence against invading pathogens. In *Caenorhabditis elegans* , the oomycete recognition response (ORR) is a transcriptional programme that provides protection against lethal infections by oomycetes, such as *Myzocytiopsis humicola* . The protein tyrosine kinase OLD-1 and the pseudokinase FLOR-1 are essential for activation of this immune response, however, the underlying molecular mechanism is poorly defined. Here, we show that FLOR-1 is regulated by the kinase-specific HSP- 90 co-chaperone CDC-37. Following tandem mass tag-based proteomics, we reveal that FLOR-1 interacts with CDC-37 and that disruption of the CDC-37/HSP-90 complex abolishes ORR gene induction. CDC-37 is required for membrane association of FLOR-1 and mediates its pathogen recognition-induced relocalisation from the plasma membrane to the cytosol, where downstream immune signalling is initiated. Surprisingly, the related active protein tyrosine kinase OLD-1 functions independently of CDC-37. Functional dissection of FLOR-1 localisation demonstrates that membrane-associated FLOR-1 is required for pathogen recognition, whereas cytosolic FLOR-1 is sufficient to activate ORR gene expression. Together, these findings uncover an unexpected role for the CDC-37/HSP-90 complex in regulating innate immunity likely through spatial control of a membrane-associated pseudokinase and reveal a chaperone-mediated mechanism that links pathogen detection to immune activation.

## Full Text Availability

The license terms selected by the author(s) for this preprint version do not permit archiving in PMC. The full text is available from the preprint server.

